# Current Practices in Myocardial Perfusion Scintigraphy in Brazil and Adherence to the IAEA Recommendations: Results of a Cross-Sectional Study

**DOI:** 10.5935/abc.20180023

**Published:** 2018-02

**Authors:** Carlos Vitor Braga Rodrigues, Anderson Oliveira, Christiane Cigagna Wiefels, Maurício de Souza Leão, Cláudio Tinoco Mesquita

**Affiliations:** 1 Setor de Medicina Nuclear - Hospital Universitário Antônio Pedro (HUAP) - Universidade Federal Fluminense (UFF), Niterói, RJ - Brazil; 2 Comissão Nacional de Energia Nuclear, Rio de Janeiro, RJ - Brazil

**Keywords:** Nuclear Medicine / methods, Myocardial Perfusion Imaging, Myocardial Ischemia / diagnostic imaging

## Abstract

**Background:**

Data on the current situation of nuclear medicine practices in cardiology in Brazil are scarce. The International Atomic Energy Agency (IAEA) has recommended eight "good practices" to minimize patients' ionizing radiation exposure during myocardial perfusion scintigraphy (MPS).

**Objectives:**

To assess the adoption of the eight good practices in MPS in Brazil.

**Methods:**

Cross-sectional study with data obtained by use of a questionnaire. All hypothesis tests performed considered a significance level of 5%.

**Results:**

We observed that 100% of the nuclear medicine services (NMS) assessed do not use thallium-201 as the preferred protocol. Regarding the use of technetium-99m, 57% of the NMS administer activities above the threshold recommended by the IAEA (36 mCi) or achieve an effective dose greater than 15 millisievert (mSv). The abbreviated stress-only myocardial perfusion imaging is not employed by 94% of the NMS; thus, only 19% count on strategies to reduce the radioactive doses. Approximately 52% of the NMS reported always performing dose adjustment for patient's weight, while 35% administer poorly calculated doses in the one-day protocol.

**Conclusion:**

A considerable number of NMS in Brazil have not adopted at least six practices recommended by the IAEA. Despite the difficulties found in nuclear practice in some Brazilian regions, almost all obstacles observed can be overcome with no cost increase, emphasizing the importance of developing strategies for adopting "good practices" when performing MPS.

## Introduction

Myocardial perfusion scintigraphy (MPS) is a non-invasive, safe technique that uses physical or pharmacological stress to detect the presence of ischemia, assessing its early changes. The complication rate of MPS does not exceed that of exercise testing, whose mortality is estimated at 0.01%.^1^

Patients with ischemia evidenced on MPS are at higher risk for adverse outcomes as compared to those with a normal test. That stratification is fundamental, because invasive approaches are only beneficial to patients at increased risk. According to the European guidelines on revascularization, the best-established techniques for diagnosing ischemia are MPS and stress echocardiography.^2^ Appropriate use of invasive procedures is fundamental, because they have a high cost. The IMPACT Study has shown that most of the cost to manage coronary disease derives from invasive procedures.^3^

Myocardial perfusion scintigraphy is the nuclear medicine procedure most used in Brazil, accounting for 54% of all scintigraphies performed within the Brazilian Unified Health System (SUS).^4^ Although widely used, the practices are heterogeneous and can be refined, especially because they employ ionizing radiation, which, by principle of radioprotection, should be used in a justified and optimized way. Santos et al.,^5^ assessing the use of scintigraphy in SUS, have observed a 12% rate of inappropriate use. Those authors have reported that, with appropriate use, there will be an 18.6% reduction in budget costs, in addition to a reduction in unnecessary radiation exposure.^5^ Oliveira et al.,^6^ however, assessing the MPS use at another institution, have found a rate of inappropriate tests of only 5.2%.^6^

Considering the heterogeneous use and radiation exposure, the International Atomic Energy Agency (IAEA) recommends eight "good practices" to minimize radiation exposure during MPS.^7^ The INCAPS Study has assessed the adoption of those practices at 308 nuclear medicine services (NMS) in 65 countries, and only 142 NMS (45%) have shown a satisfactory rate. So far, there are no data on the use of those recommendations in Brazil, which is this study's objective.

## Methods

This is a cross-sectional study with online self-administered questionnaire, which was sent to the email address of the technical managers of the NMS in operation in Brazil (403 NMS on the first trimester of 2016, according to data obtained at the site of the Brazilian Committee of Nuclear Energy (CNEN). The inclusion criterion in the study was that the NMS must be authorized by the CNEN to operate. The NMS performing fewer than 20 MPS per month, as well as duplicated responses, were excluded from this study, which resulted in 63 respondents (16% of total).

The questionnaire was elaborated based on the North American and European guidelines, with questions selected from the following IAEA publications: *Quality Management Audits in Nuclear Medicine Practices* (QUANUM)^8^ and *Nuclear Medicine Database* (NUMDAB).^9^ The questionnaire consisted of 49 questions, divided into the following 7 domains: demographic data of the NMS (5 items); technical team (10 items); patient care (4 items); radiopharmacy (8 items); equipment (7 items); test protocol (20 items); and postprocessing and image interpretation (2 items).

The multidisciplinary team of the NMS was considered to be complete when having at least one professional of each category: nuclear physician, medical physicist, pharmacist, biomedical physician scientist, nurse and technician.

Quality index (QI) was adopted to measure objectively the quality of the MPS, and comprises the sum of the practices that can be adopted in an NMS. The QI ranges from 0 to 8, a QI ≥ 6 being considered the desirable level for an NMS to have as suggested by the IAEA.^7^

### Statistical analysis

The variables were tested for normality by use of the Kolmogorov-Smirnov method, revealing a non-normal distribution. Thus, descriptive analysis was performed by use of medians and interquartile range, and the Kruskal-Wallis and the Mann-Whitney U tests for independent samples were used. The Statistical Package for the Social Sciences*,* version 21, was used for the statistical analysis. All hypothesis tests performed considered a significance level of 5%, that is, the null hypothesis was rejected when p value < 0.05.

## Results

The responding 63 NMS reflect the practice of 972 professionals, who account for an estimate of 13,200 MPS per month.

**Figure 1:**
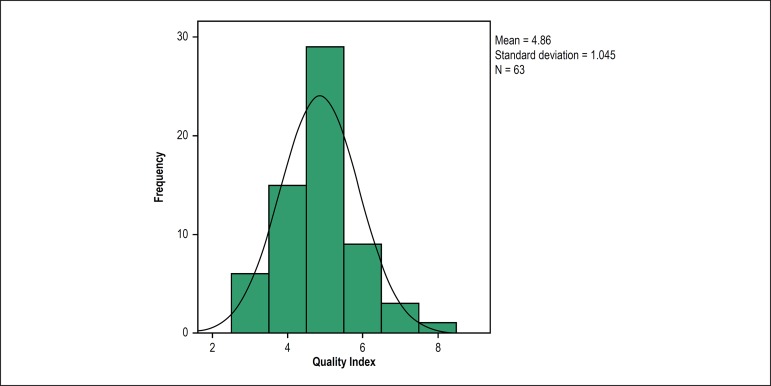
Distribution of the quality index (0 to 8) of good practices of 63 nuclear cardiology services in Brazil, 2016.

Figure 1 shows the histogram of the QI distribution at 63 NMS in Brazil, where the median QI found was 5. The lowest QI was 3, the lowest quartile equivalent to 25% of the QI scores was 4, and the highest quartile was 5. A QI ≥ 6, which is the desirable index, was only observed in 13 NMS (20.6% of the sample).

Table 1 discriminates the QI values according to the major characteristics of the NMS, aiming at identifying those associated with the highest QI values. Two variables showed significant association with an elevated QI: 1) the NMS location inside academic institutions as compared to non-academic ones (p = 0.046); and 2) presence in the NMS of a complete multidisciplinary team as compared to absence thereof (p = 0.030).

**Table 1:** Quality index according to the demographic, professional and regional characteristics of nuclear medicine services (NMS)

	N	Mean	Median	Standard deviation	p value
**Brazilian region**					
Southeast	34	5	5	1.044	0.750*
South	17	4.76	5	1.200	
West-Central	2	5.00	5	0.000	
Northeast	8	4.50	4	0.926	
North	2	4.50	4	0.707	
**Type of NMS**					
Private	55	4.91	5	1.076	**0.329^ƚ^**
Public	8	4.50	5	0.756	
**University-affiliated**					
Yes	7	5.57	5	0.78	
No	56	4.77	5	1.04	
**> 3 nuclear physicians**					
Yes	45	4.76	5	0.85	**0.204^ƚ^**
No	16	5.19	5	1.47	
**Complete multidisciplinary team**					
Yes	12	5.33	5	0.98	**0.030^ƚ^**
No	51	4.75	5	1.03	
**Exclusive NMS**					
Yes	19	4.86	5		**0.956^ƚ^**
No	44		5		

* Independent-Samples Kruskal-Wallis test;

ƚ Independent-Samples Mann-Whitney U test

When assessing the amount of MPS performed monthly and its relation to the desirable QI (Table 2), we observed that institutions with QI ≥ 6 performed a statistically higher number of MPS than those that did not adopt at least six good practices (p = 0.043).

**Table 2:** Comparison of the mean numbers of myocardial perfusion scintigraphy (MPS) performed at the 63 nuclear medicine services

	N	Mean	Median	Standard deviation	p value
**Number of MPS per month**					
≥ 6 Good practices	13	298	280	230	0.043*
< 6 Good practices	50	186	120	304	

* Mann-Whitney U test

When assessing the presence of each good practice in the 63 NMS (Table 3), the most frequently adopted by all were as follows: 1) do not use the thallium-stress protocol; 2) do not use the dual-isotope protocol; and consequently 3) do not use increased Tl-201 activities. Conversely, the least frequently adopted good practice was the use of the abbreviated stress-only myocardial perfusion imaging, in only 6% of the NMS.

**Table 3:** Frequency (%) of the adoption of each good practice at the nuclear medicine services assessed in Brazil, 2016

Good practices	Brazil	
A	63	(100)
B	63	(100)
C	27	(42.86)
D	63	(100)
E	4	(6.35)
F	12	(19.05)
G	33	(52.38)
H	41	(65.08)

A: Avoid thallium-stress protocol; B: Avoid dual-isotope protocol; C: Avoid high Tc-99m activities; D: Avoid high Tl-201 activities; E: Perform only "Stress-Only"; F: Use strategies focused on dose reduction; G: Patient's weight-based activities; H: Avoid inappropriate activities that can generate the shine-through artifact.

## Discussion

The IAEA has been dedicated to promoting good practices in nuclear cardiology, undertaking the largest research about cardiological tests so far, by use of a cross-sectional study of global comprehensiveness called INCAPS, which evidenced that the adoption of good practices in NMS is highly heterogeneous in the continents. The NMS in Asia and Latin America showed the worst performance, with less than one quarter of the NMS achieving the desirable QI (≥ 6 good practices).^7^ Information on the situation of the NMS in Brazil is scarce. After that research, the Brazilian Society of Nuclear Medicine, concerned with qualified practice, was one of the first entities to endorse the adoption of good practices in its guidelines, aimed at the continuous search for a reduction in radiation exposure (optimization).^10^

Thallium-201 scintigraphy has unfavorable physical characteristics, such as low counting rate and long physical half-life, which are associated with a higher dose of radiation absorbed, being considered a second option to Tc-99m-sestamibi. The use of Tl-201 is strictly recommended for myocardial viability studies, but with the new devices with highly effective detectors, there is a renewed interest in ultrafast dual-isotope protocols that enable the use of low doses and conveniently allow performing the complete test in less than 30 minutes.^11^ In our study, we observed that 100% of the NMS assessed used neither Tl-201 nor dual-isotope as the preferred protocol, which is a good practice also associated with the financial aspect, considering the lower cost of Tc-99m-sestamibi and its easy use, which involves a medication kit. Thus, currently the traditional protocols of one or two days still predominate.

Conversely, the least frequently adopted good practice by the NMS in our study was the abbreviated stress-only myocardial perfusion imaging.^12^ Chang et al.^13^ have demonstrated that it is safe to use a single stress phase, without rest, in normal tests from the perfusional and contractile function viewpoint, which facilitated the dynamics of the NMS and reduced by 61% the use of radiopharmaceuticals and radiation exposure. Gowd et al.^14^ have listed the limitations to its wide adoption, such as non-familiarity with the assessment of a single phase, the need for processing images immediately after their acquisition, and the issues regarding reimbursement of expenses, considering that a significant part of the test is paid with the resting phase. Oliveira et al.^15^ have been the first to approach the use of that protocol in Brazil, but the experience is still incipient.

An accurate test requires the use of proper radiation doses, avoiding the "shine-through" phenomenon.^16^ One third of the NMS assessed still administer doses that can allow the interference of residual radiation with later images in the one-day protocol.^17^ In that protocol, respecting the minimum three-hour interval between the phases, a dose three times higher than that of the first phase is required to avoid that artifact, which can lead to a reduction in the ischemic burden or even to false-negative results.^16^ Recent studies have shown that protocols with ultra-low doses of sestamibi (5 mCi) during stress can be even more appropriate to prevent that artifact.^17^

The IAEA has suggested the Tc-99m threshold of 36 mCi as the maximum activity to be administered in a single injection;^7^ however, half of the NMS assessed use activities over that threshold. Such thresholds are usually exceeded when the patient has a high body weight, the best strategy for that patient being to undergo MPS in the two-day protocol, eliminating, thus, the need for tripling the dose, providing lower radiation exposure and higher image quality.^10^ The adjustment of the dose for the patient's weight is part of the CNEN norms and should be adopted as a rule.^18^ Nevertheless, almost half of the NMS assessed have not adopted routinely this practice, missing an opportunity for improvement. That adjustment is aimed at using appropriate radiation doses to each patient's weight and attenuation rate, preventing overexposure or insufficient exposure, which leads to a low quality test.^19^

In addition, strategies for dose reduction have been considered. There is high-technology hardware, such as CZT cameras,^20,21^ which provide high image resolution, and hybrid devices, such as SPECT-CT, which can eliminate the attenuation of soft tissues,^22^ but they are not widely available. A strategy that can be used without additional costs for those without attenuation correction is the prone position during the acquisition of the stress phase of MPS. Placing the patient in the prone position reduces diaphragmatic attenuation and its interference with the images.^23,24^ Many NMS have reported using that technique, but that can only be considered a strategy of dose reduction if the single stress phase is a practice adopted by the entire NMS. In prone MPS, the stress phase shows normal perfusion aspect and preserved contractility, but the patient should undergo the second phase anyhow. There was no dose reduction during that process.

In general, the QI was significantly higher in the academic institutions. In 2010, the MPS performed inside university-affiliated hospitals showed more appropriate and precise indications.^25^ The NMS that promote research are constantly searching for knowledge, being updated by recent studies and new international recommendations very fast, being always one step ahead.

Another important and innovative finding was the significantly higher QI of the institutions that count on a complete multiprofessional team, comprised by nuclear physician, medical physicist, pharmacist, biomedical physician scientist, nurse and technician (at least one of each). Thus, the NMS with professionals of different areas provide better patient care by adding multiple domains of knowledge.

The present study has some limitations, the most evident being the self-administered questionnaire, which attributes to the respondent the research's degree of reliability. Despite being a random sample, most respondents lived in the southern and southeastern regions of Brazil.

## Conclusion

Our study assessed the adoption of good practices in nuclear cardiology tests at NMS in Brazil. Although the response rate to the questionnaire was only 16% of the total NMS on operation, not representing a probabilistic sample, this is the largest data collection about nuclear medicine practices in cardiology in Brazil so far. We observed that the adoption rate of good practices, measured by use of the QI, is heterogeneous, showing an opportunity for improvement. One fifth of the participants has achieved excellence, which was more frequent in university-affiliated SNM and in those with a complete multidisciplinary team.

We found that the adoption of good practices in the nuclear medicine tests in cardiology by the NMS assessed in Brazil is equivalent to that of other countries in Latin America, Asia and even North America, being, however, lower than that observed in other continents.

There is the opportunity for improvement without cost increase, which requires the adoption of encouraging educational interventions to strengthen cardiology in Brazil.
